# Self-powered electrotactile textile haptic glove for enhanced human-machine interface

**DOI:** 10.1126/sciadv.adt0318

**Published:** 2025-03-21

**Authors:** Guoqiang Xu, Haoyu Wang, Guangyao Zhao, Jingjing Fu, Kuanming Yao, Shengxin Jia, Rui Shi, Xingcan Huang, Pengcheng Wu, Jiyu Li, Binbin Zhang, Chun Ki Yiu, Zhihao Zhou, Chaojie Chen, Xinyuan Li, Zhengchun Peng, Yunlong Zi, Zijian Zheng, Xinge Yu

**Affiliations:** ^1^ Department of Biomedical Engineering, City University of Hong Kong, Kowloon Tong, Hong Kong SAR, China.; ^2^ Department of Mechanical and Automation Engineering, The Chinese University of Hong Kong, Shatin, N.T., Hong Kong SAR, China.; ^3^ Department of Applied Biology and Chemical Technology, Faculty of Science, The Hong Kong Polytechnic University, Hong Kong SAR, China.; ^4^ Hong Kong Center for Cerebro-Cardiovascular Health Engineering, Hong Kong Science Park, New Territories, Hong Kong SAR, China.; ^5^ Beijing Institute of Nanoenergy and Nanosystems, Chinese Academy of Sciences, Beijing 100083, China.; ^6^ School of Electronic Information and Electrical Engineering, Shanghai Jiao Tong University, Shanghai 200240, China.; ^7^ Thrust of Sustainable Energy and Environment, The Hong Kong University of Science and Technology (Guangzhou), Nansha, Guangzhou, Guangdong 511400, China.; ^8^ HKUST Shenzhen-Hong Kong Collaborative Innovation Research Institute, Futian, Shenzhen, Guangdong 518048, China.; ^9^ Research Institute for Intelligent Wearable Systems (RI-IWEAR), The Hong Kong Polytechnic University, Hong Kong SAR, China.; ^10^ Research Institute for Smart Energy (RI-RISE), The Hong Kong Polytechnic University, Hong Kong SAR, China.; ^11^ Institute of Digital Medicine, City University of Hong Kong, Hong Kong SAR, China.; ^12^ City University of Hong Kong Shenzhen Research Institute, Shenzhen, Guangdong, 518057, China.

## Abstract

Human-machine interface (HMI) plays an important role in various fields, where haptic technologies provide crucial tactile feedback that greatly enhances user experience, especially in virtual reality/augmented reality, prosthetic control, and therapeutic applications. Through tactile feedback, users can interact with devices in a more realistic way, thereby improving the overall effectiveness of the experience. However, existing haptic devices are often bulky due to cumbersome instruments and power modules, limiting comfort and portability. Here, we introduce a concept of wearable haptic technology: a thin, soft, self-powered electrotactile textile haptic (SPETH) glove that uses the triboelectric effect and gas breakdown discharge for localized electrical stimulation. Daily hand movements generate sufficient mechanical energy to power the SPETH glove. Its features—softness, lightweight, self-sustainability, portability, and affordability—enable it to provide tactile feedback anytime and anywhere without external equipment. This makes the SPETH glove an enhanced, battery-free HMI suitable for a wide range of applications.

## Teaser

A self-powered electrotactile glove based on the triboelectric effect and discharge is developed to enable electrical stimulation.

## INTRODUCTION

In recent years, the rapid development of virtual reality (VR)/augmented reality (AR) and metaverse has made a revolutionary impact on the interaction between humans and machines (*1*–*5*). These emerging technologies establish an interactive mode between users and physical/virtual objects, which includes information of visual (*6*–*8*), auditory (*9*, *10*), tactile (*11*), olfactory (*12*, *13*), and other senses (*14*, *15*). Among these senses, tactile feedback, also known as haptic, plays an important role in transmitting and reproducing “touch,” which can enhance VR/AR experience and improve accuracy in interacting with machines and synchronizing information from the robotic end to the user end (*16*–*18*). Therefore, haptic technologies are the key for enhancing interactions in the human-machine interface (*19*).

The state-of-the-art haptic technologies basically associate with mechanical vibration, pneumatic devices, and electrical stimulation, which directly provide stimulus to the mechanoreceptors under the skin for achieving perception (*20*, *21*). These devices usually require numerous additional accessories and cables for power transmission and connectivity, resulting in large, heavy equipment, thus notably limiting their potential applications (*22*–*24*). Besides, the challenges stemming from heat generation, high working voltage, and high costs of these devices also pose concerns in safety issue and universal practical use (*25*). Focusing on solving these issues, recent efforts on the development of thin and soft wearable haptic interface have been made by introducing new stimulation formats (*11*, *16*, *26*, *27*), adopting innovative electrode materials (*28*–*30*), improving the working efficiency of mechanical actuators (*1*, *2*, *31*), and implanting radio frequency as a power source (*16*, *20*, *32*). However, the issues in the power management from power consumption to physical demission remain.

It would be revolutionary for haptic technologies to realize self-powered machinery, such as self-powered sensors (*33*–*37*). It is well known that sensors typically consume much less power than actuators. To realize self-powered haptic devices, it is important to choose high-efficiency power conversion technologies as well as lower the power consumption of the actuators (*28*, *38*). Among all the self-powered sensing technologies, triboelectric nanogenerators (TENGs) seem to be the most suitable in providing high voltages from transducing mechanical forces. TENGs have been proven to be useful as pacemakers and to excel due to their inherent discharge properties (*39*–*41*). This unique ability to generate discharges can potentially be harnessed as a method for tactile perception. Thus, it may be possible to develop a self-powered haptic interface by formulating a proper TENG-based electrical stimulation strategy.

Here, we report self-powered wearable haptic technology by adopting TENG as an energy-generating source to provide an electrotactile tool, i.e., a thin, soft, comfortable-to-wear self-powered electrotactile textile haptic (SPETH) glove. The SPETH glove enables self-powered tactile reproduction by electrical stimulation through the triboelectric discharge effect–induced high-frequency alternating current (*42*–*44*). The mechanism of triboelectrification associating with triboelectrification, electrostatic induction, and breakdown discharge (*45*) allows the conversion of hand motion–induced mechanical energy into large impulse current (0.2 to 4.8 mA). This impulse current can effectively reduce skin impedance and then serve as a controllable electrotactile means to the mechanoreceptors under the skin for tactile perception. By controlling the energy released, the feeling intensities of tactile sensation can be adjusted. Therefore, the SPETH glove can be used for a broad range of applications, from VR/AR, to robotic interaction, to prosthetic control/sensing, and many others.

## RESULTS

### Concept and design of the SPETH glove

**Fig. 1.**
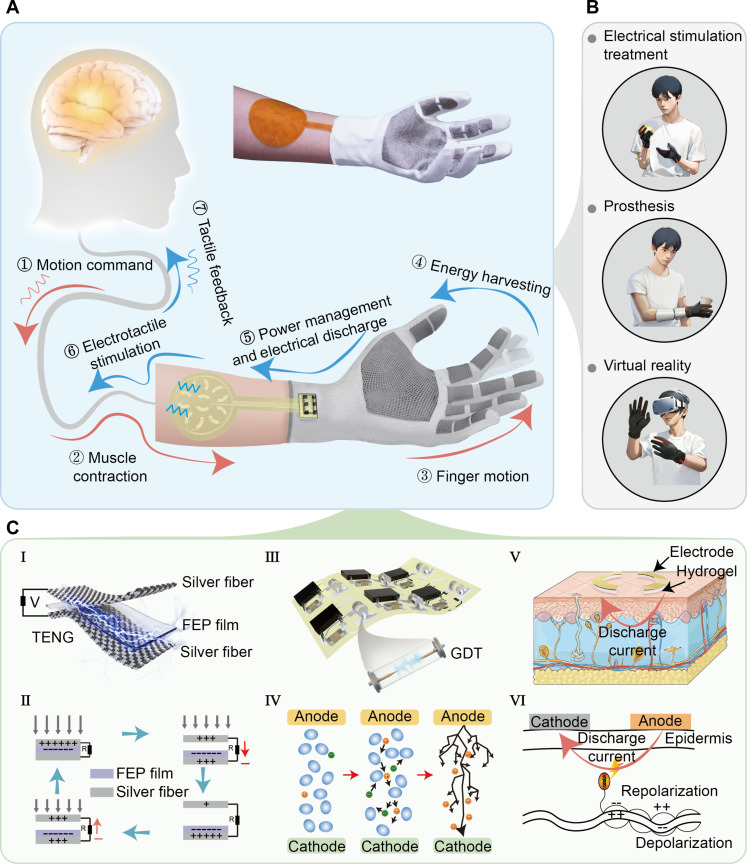
Schematic diagram of triboelectric effect and discharge current–enabled SPETH glove. (**A**) Illustration of the SPETH glove. (**B**) Applications of the SPETH glove, including electrostimulation therapy, prosthetics, and virtual reality. (**C**) Components and schematic diagram of the haptic glove, including [(C), I] textile-based TENG unit and [(C), II] working principle, [(C), III] schematic of management circuit with gas discharge tube and [(C), IV] principle of air discharge, where the oval-shaped diagrams represent neutral particles in the air, while the circles with positive and negative signs correspond to positively and negatively charged ions, and [(C), V] skin electrodes and [(C), VI] principle of haptic sensation.

To enhance interactive perception applications between user and machines, the SPETH glove is designed to provide users haptic feedback through a self-powering mechanism. Figure 1A shows the schematic diagram of the operational mechanism and the applications of the SPETH glove, where a self-powered electrotactile interaction is achieved through the triboelectric discharge effect. In detail, when considering a daily physical activity, the human brain initiates a motion command, resulting in muscle contraction and ensuing finger movements. Subsequently, the SPETH glove worn on the hand collects electrostatic energy through physical contact with objects, storing this harvested energy within a power management circuit. Once the energy accrues to a critical threshold, a breakdown discharge happens in the gas discharge tube (GDT), which is integrated within the management circuit. This discharge process enables a discharge current into the skin through meticulously positioned electrodes, which then stimulate the sensory receptors in the skin and elicit neural excitation. Afterward, this excitation propagates through the peripheral nerves and ultimately reaches the brain, where it is assimilated as tactile feedback, completing the sensory loop in a coherent and responsive manner. Besides, the trigger motion can be given not only by human motion but also by mechanical motions from robotic hands, prosthetics, and other machines. Such interaction effectively bridges the gap between humans and diverse mechanical interfaces, establishing a self-powered bidirectional sensing and feedback system between them. Its envisioned utility spans a diverse spectrum of applications including electrostimulation therapy, advanced prosthetic feedback mechanisms, and cutting-edge VR/AR experiences (Fig. 1B).

Figure 1C shows the detailed design of the SPETH glove, consisting of three key components: a textile-based triboelectric unit, a management circuit with the GDT, and a skin electrode. Specifically, the textile-based triboelectric unit is based on a three-layer design, where the top electrode is crafted with conductive silver fiber intricately embroidered with the fabric. The bottom layer entails affixing a fluorinated ethylene propylene (FEP) film to the conductive silver fiber on the fabric, culminating in a comprehensive TENG unit (Figure 1C-I). It is important to highlight that the FEP film we used is a commercial product with a single-sided adhesive, enabling direct attachment to the bottom fabric electrode, which works in conjunction with the top fabric electrode to form a TENG unit. Given the inherent loose structure of the fabric, a completely tight contact between the FEP film and the top fabric electrode is generally not achieved. Instead, when pressure is applied, the gap between the two electrodes changes, producing voltage through electrostatic induction. The seamless embedding of conductive silver fiber into textile materials can effectively merge the traditional craft of embroidery with contemporary electronic functionality. This integration not only simplifies the manufacturing process and enhances aesthetics, but also improves the overall efficiency and reliability of the TENG unit. Leveraging the advantages of embroidery technology not only allows for the creation of intricate and precise patterns for the TENGs but also enables good flexibility and lightweight characteristics for the wearable devices. The resulting textile-based TENG unit exhibits excellent triboelectric performance, allowing it to be an ideal choice as a self-powered source. The detailed embroidery process and product image are illustrated in fig. S1 and movie S1.

On the basis of the working principle of triboelectrification and electrostatic induction, an alternating high-voltage output can be generated in the compressed and released process of the SPETH glove (Fig. 1C-II) (*46*). The power management circuit of the SPETH glove incorporates a GDT, as shown in Fig. 1C-III. This circuit is essential for effective energy management and ensuring the device’s safety. Among them, energy harvested by the textile-based TENGs is stored in a capacitor of the power management circuit. If this voltage surpasses the activation threshold of the GDT, it triggers an avalanche breakdown discharge (*47*). This avalanche breakdown process begins when the intense electric field generated by the high voltage ionizes the gas molecules encapsulated within the GDT. Ionization occurs when the electric field is strong enough to impart enough energy to some gas molecules, causing them to lose electrons and form positive ions and free electrons. As ionization occurs, free electrons can collide with other gas molecules, leading to further ionization (Fig. 1C-IV). This cascading series of events creates an increase in the number of free charge carriers (ions and electrons) within the gas, much like an avalanche (*48*). As a result, a pulse current is generated for delivering a tangible stimulus to the user (Fig. 1C-V). Last, as highlighted by Fig. 1C-V, the skin electrodes designed for tactile feedback are composed of two semicircular electrodes overlaying a 1-mm-thick hydrogel to enhance good contact with the skin with decreased impedance. It is worth noting that the addition of hydrogel is necessary, which can greatly reduce skin impedance and thus enhance sensory sensitivity (fig. S2). Figure 1C-VI shows the principle behind the generation of haptic sensations, which associates with a direct and intuitive interaction between the device and the user. These components enable a self-sustaining system, providing haptic sensations through a passive self-powered manner without additional battery or external cables. Specifically, when the electrotactile haptic device administers electrical currents to localized skin areas, it triggers nearby mechanoreceptors’ axons to generate action potentials following established neural pathways (*49*). These signals are subsequently transmitted to the somatosensory cortex, where the brain interprets them as tactile sensations (*50*).

### Electrical performance of the triboelectric unit in the SPETH glove

**Fig. 2.**
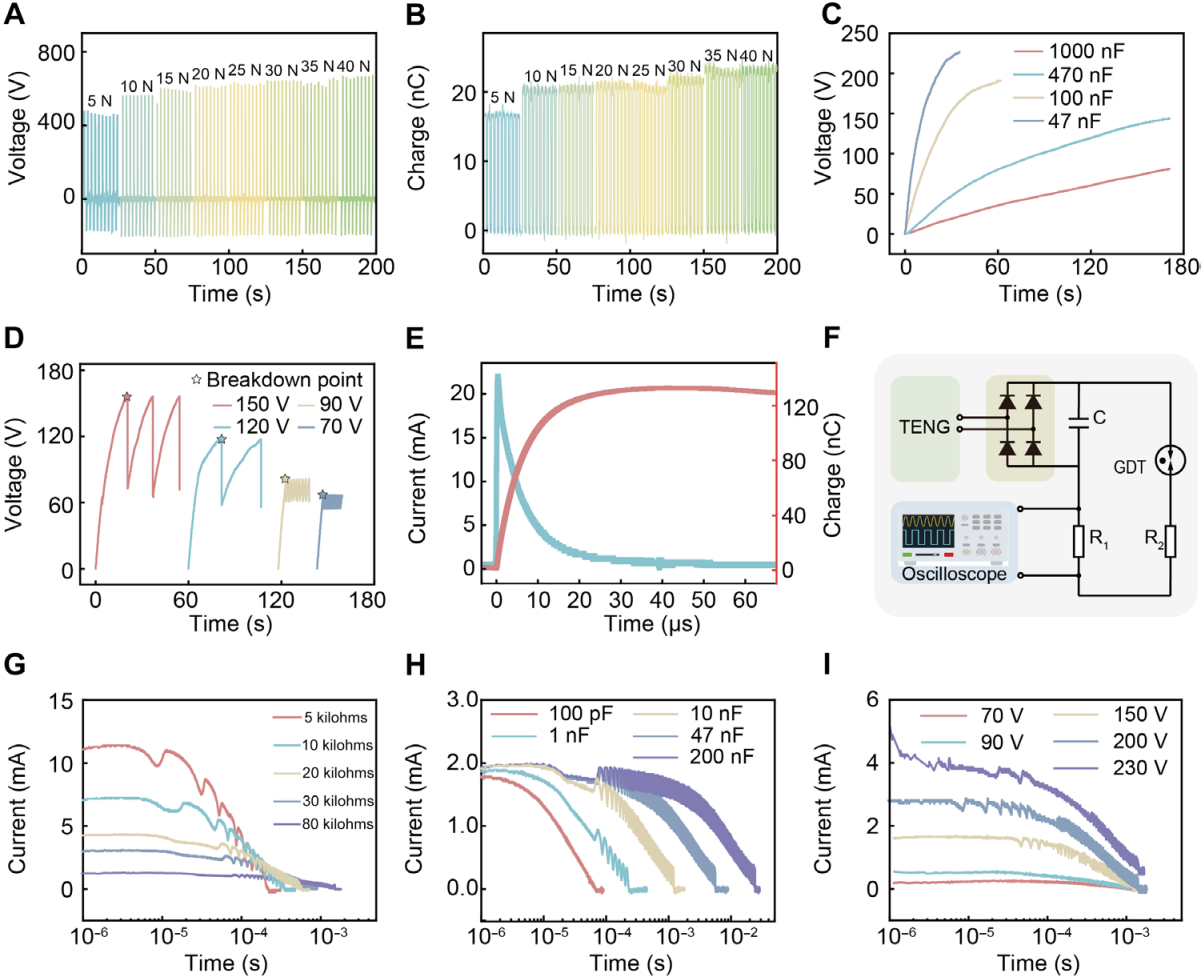
Electrical performance of SPETH glove and discharge current. (**A**) Voltage and (**B**) charge curves of the TENG unit of SPETH glove under different pressures, where the size of TENG is 1.5 cm by 1.5 cm. (**C**) Voltage-time curve of charging different capacitors using the TENG unit. (**D**) Voltage-time curve of GDT under different voltage breakdown thresholds. (**E**) Current and charge curves during skin discharge. (**F**) Schematic diagram of current testing for GDT discharge. Current curves of different GDTs under different (**G**) resistances, (**H**) capacitances, and (**I**) discharge thresholds.

To evaluate the output performance of the triboelectric unit of the SPETH glove, comprehensive experiments have been designed to characterize the TENG device. As shown in Fig. 2 (A and B) and fig. S3, both the output voltage and induced charges increase with the increase of loading pressure, while the increasing trend of the electrical performance of the TENG slows down and is lastly saturated as pressure increases. It can be found the output voltage and charge output approach saturated at ~600 V and ~20 nC, respectively, under 160 kPa. Upon initial application of pressure, the output rapidly increases due to the deformation of the textile electrode, effectively increasing the actual contact area. However, at higher pressures, the deformation eventually reaches its elastic limit at a specific pressure (*51*). As a result, the output tends toward saturation, ultimately yielding an output of approximately 250 V and 400 nC with each grasp or tap. Figure 2C shows the charging curves of capacitors of various sizes using the SPETH glove, indicating its strong output capability and ensuring reliable subsequent electrical stimulation. It only takes approximately 60 s to charge a 100-nF capacitor to 200 V, and for smaller capacities, the charging time is even shorter.

Traditional TENGs are commonly made of rigid materials to achieve a larger contact area and input force that can generate voltages exceeding several thousand volts, which can easily lead to breakdown discharge (*52*, *53*). However, wearable TENG devices often produce lower outputs limited by applied flexible materials and restricted contact force and form (*54*), necessitating the generation of pulse currents at lower voltages for optimal functionality. Typically, achieving pulsed currents requires the use of switches, and we compared several commonly used options, including metal-oxide semiconductor field-effect transistors, electrostatic switches, and electromagnetic relays (table S1). GDTs effectively operate under the specific constraints of TENGs, requiring low energy for activation, generating strong pulsed currents, and simplifying the integration process without the need for additional control circuitry. These advantages make GDTs an optimal solution for our research objectives. In conclusion, we believe that the GDT is the most viable option for our design. This phenomenon is exemplified in Fig. 2D, where gas breakdown sites are highlighted with stars. When the GDT reaches its breakdown voltage (*V*_b_), it breaks down and opens a channel, facilitating current conduction. As the voltage across the capacitor gradually decreases, it eventually reaches the extinction voltage (*V*_e_), triggering the closure of the GDT channel and halting current flow. Subsequent recharging of the capacitor occurs until the next breakdown event. The initial charging process takes longer, which starts from zero voltage, whereas subsequent recharges take much less time. Consequently, storage capacitors can achieve breakdown threshold voltage much faster, enhancing process efficiency. For instance, current and charge curves during skin discharge with a GDT are illustrated (Fig. 2E). The experimental setup features a GDT with a breakdown voltage of 120 V and a 10-nF rated storage capacitor. It takes 1200 nC of charge to charge the capacitor to the breakdown threshold, but considering the actual current flowing through the skin, it is found that the total transferred charge is only about 120 nC, which is much lower than the total stored charge. This discrepancy occurs because the stored charge in the capacitor is not entirely released during discharge, thereby reducing the threshold for subsequent electrical breakdowns, facilitating GDT retriggering. Figure S4 shows additional data on skin discharge that can further elucidate this phenomenon. Despite the high actual current generated in a short exposure time, no skin damage is inflicted, as depicted in fig. S5.

Figure 2F shows the circuit diagram of inducing breakdown discharge in the GDT by using TENG, which is used to assess the discharge performance of the GDT, in this circuit, alternative current generated by the TENG is rectified into direct current, subsequently stored in capacitor *C*. The setup involves the serial connection of the GDT and resistor *R*_2_ (representing skin impedance), followed by parallel connection with *C*. Here, another resistor, *R*_1_ (~3.9 kilohms), is used as a minor resistor for monitoring the discharge current. Because the value of *R*_1_ is lower than the oscilloscope’s internal resistance of 100 megohms, it can provide accurate sensing signals. Upon reaching the breakdown threshold voltage across the GDT, the discharge current flows through *R*_2_ and *R*_1_, effectively stimulating the skin. Throughout the system, the value of the applied capacitor *C*, the skin’s equivalent impedance, and the threshold of the GDT all influence the actual output current. On the basis of this circuit, the major influential factors, including skin impedance, capacitance, and discharge threshold, were studied, as shown in Fig. 2 (G and H). Among them, Fig. 2G illustrates the impact of equivalent skin impedance under a fixed capacitance of 10 nF and a breakdown voltage of 150 V, which could generate a very apparent situation on skin. The influence of capacitance *C* is explored in Fig. 2H with a constant resistance of 50 kilohms (approximately equal to the human body impedance) and a breakdown voltage of 150 V. Figure 2I shows the effect of the breakdown voltage with a fixed capacitance of 47 nF and a resistance of 50 kilohms. The observed trend in the data aligns with Ohm’s law, showing that a decrease in skin impedance leads to an increase in discharge current. The duration of the discharge current is directly influenced by capacitance, and raising the breakdown threshold typically results in higher discharge currents.

### Haptic feedback of the SPETH glove

**Fig. 3.**
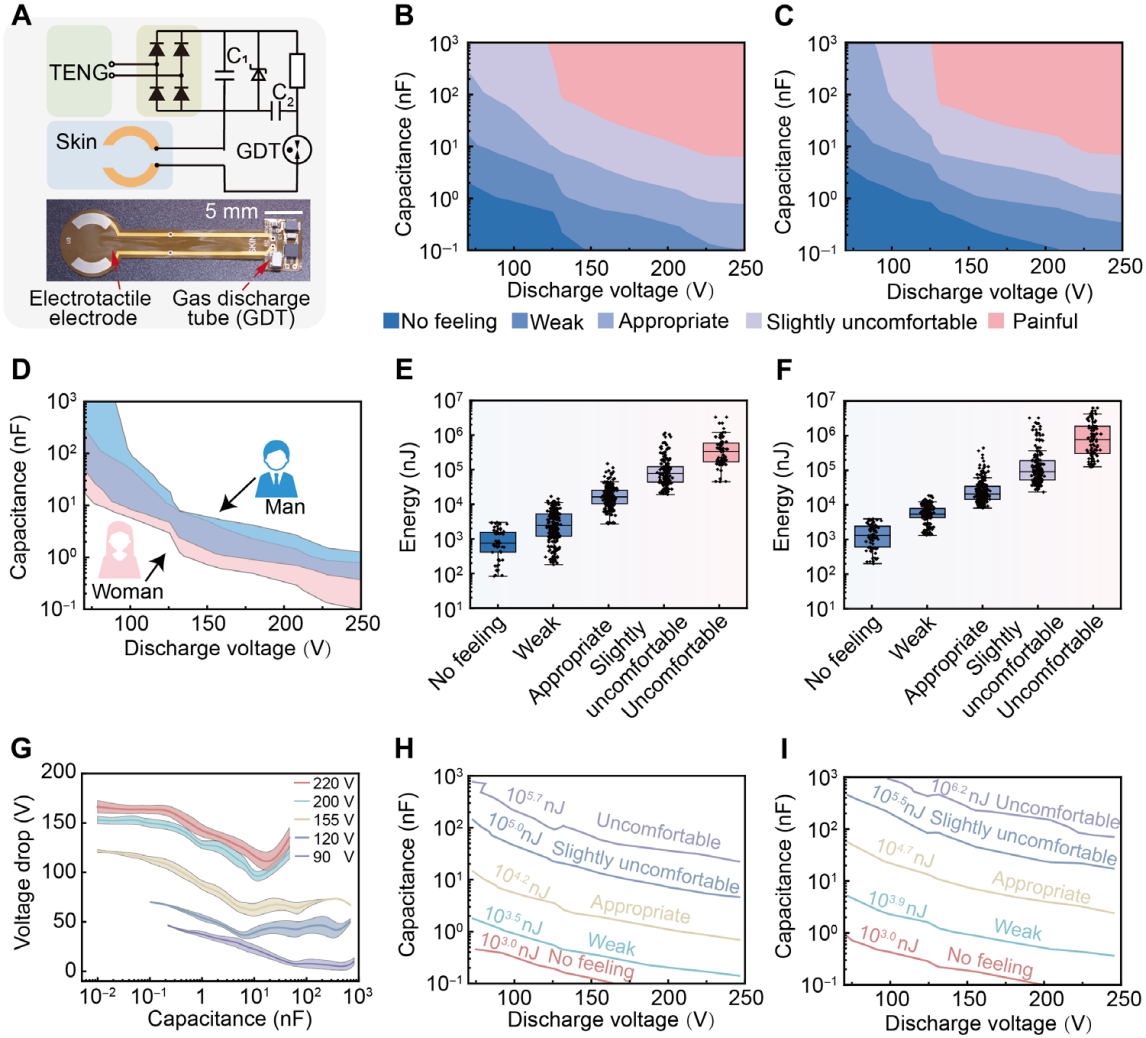
Results display of electric stimulation tactile feedback of SPETH glove. (**A**) Schematic diagram of tactile feedback testing. Grading chart of sensations for (**B**) female and (**C**) male participants at different capacitances and discharge threshold voltages. A total of 30 participants were included, and the sensations were classified into five levels: no feeling, weak, appropriate, slightly uncomfortable, and painful. (**D**) Comparison graph of comfort zones for male and female participants. Male is represented by blue, while female is represented by pink. (**E**) Voltage drop curve of GDT at different discharge thresholds and capacitances. Boxplot of electric stimulation energy received on the skin for (**F**) female and (**G**) male participants at different sensation levels. Distribution graph of electric stimulation energy for (**H**) female and (**I**) male participants at different sensations. The electric stimulation energy is defined by the average values of [(F) and (G)].

The optimization of the haptic function of the SPETH glove is done through the careful design of the circuit and the selection of materials. Figure 3A shows the circuit design diagram of the haptic part in the SPETH glove. The current generated by the TENG is rectified into direct current and stored in capacitor *C*_1_. A Zener diode connects in parallel with *C*_1_ to prevent excess voltage accumulation. Subsequently, capacitor *C*_2_ is series connected with a 100-kilohm resistor, then paralleled with *C*_1_ to ensure continuous charging of *C*_2_ from *C*_1_. Therefore, *C*_2_ is connected to both the skin and a GDT for discharging electricity for electrotactile feedback. Specifically, upon reaching the breakdown voltage threshold of the GDT, the current passes through the GDT and semicircular electrodes with hydrogel to the skin, generating perceptible electrode haptic feedback. The choice of a semicircular electrode was made to create a deeper and wider current distribution area on the skin, preventing current concentration in a localized region (fig. S6). The operational dynamics of *C*_1_ and *C*_2_, including their charging and discharging cycles, are shown in fig. S7.

Key factors influencing haptic perception intensity in this system include the capacitance of *C*_2_, the GDT’s breakdown threshold, and the geometry and location of electrodes on the skin. A user study of 30 participants (15 males and 15 females) was conducted to analyze how variations in capacitance and breakdown thresholds influence haptic perception on human arms (Fig. 3, B and C). Sensations were classified into five levels: no feeling, weak, appropriate, slightly uncomfortable, and painful. Blinded tests by the participants associated with telling a level that best reflected their feelings. The data show that increasing capacitance or breakdown threshold can improve haptic perception. Moreover, it shows that gender is also an issue causing perception differences, where the data indicate that capacitance for females is notably lower than that for males, i.e., from 20 to 100 nF to <1 nF, as the breakdown voltage increases from 70 to 250 V (Fig. 3D). The perception difference is basically due to physiological differences from males to females, such as skin impedance and dermal thickness (*55*). Figure 3E shows the actual voltage drop (*V*_drop_) as a function of varying capacitances and breakdown thresholds, based on Eq. 1

$$V_{\text{drop}}=V_{b}-V_{e}$$ (1)

where *V*_b_ is the breakdown voltage of GDT and *V*_e_ is the extinction voltage of GDT. It shows that increasing capacitance leads to a gradual decrease in the voltage drop, suggesting that the actual charge passed does not increase linearly with capacitance under fixed breakdown thresholds. Hence, a high capacitance paired with a low breakdown threshold provides only a mild haptic perception due to the small amount of charge transmission through the skin. The transmitted energy passed through the skin *W* can be approximated as Eq. 2

$$W=\frac{1}{2}C\left(V_{b}^{2}-V_{e}^{2}\right)$$ (2)

Subsequently, calculations were conducted to assess the energy inputted to the skin of male and female participants within the graded zones depicted in Fig. 3 (F and G). The results show that the energy range extends over three orders of magnitude, from 1 to 10^3^ μJ, transitioning from no feeling to painful. Within this range, males typically require approximately 30 μJ of energy to elicit an appropriate sensation, whereas females only need about 10 μJ. Furthermore, in terms of sensitivity and comfortability, males generally experience discomfort when a single discharge delivers more than 1000 μJ to the skin. In contrast, females may feel discomfort at discharge energies as low as 500 μJ. The results suggest notable variability in sensory thresholds, both between genders and among individuals. It should be noted that the sensation primarily depends on the energy input rather than the size of the capacitor or the threshold of discharge voltage. Furthermore, the contour maps derived from the medians of Fig. 3 (H and I) correspond with the distribution of sensation levels, emphasizing that the vigor of tactile stimulation is predominantly contingent upon the magnitude of energy input (Fig. 3, F and G). It is noteworthy that within this framework, the energy requisite for an individual haptic perception typically operates at the microjoule level, feasibly achieved through single cycle output of the TENG, thereby ensuring the system’s sustained functionality. Besides, in practical applications, larger capacitors with lower charging voltages generally yield higher charging efficiency due to their inherent properties, as discussed in previous research on TENG charging efficiency (*56*). Therefore, to achieve equivalent stimulation effects, selecting capacitors with greater capacitance and lower discharge threshold voltages appears advantageous.

**Fig. 4.**
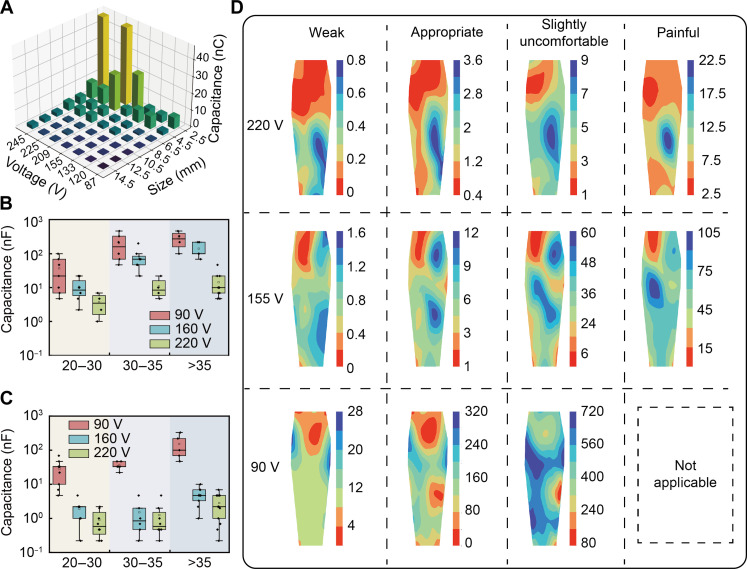
Threshold characterization results for the arm. (**A**) Comparison of voltage and capacitance for minimal tactile perception with electrodes of different sizes. (**B**) Comparison of comfortable threshold at the center of the arm for males in different age groups. (**C**) Comparison of comfortable threshold at the center of the arm for females in different age groups. (**D**) Heatmap displaying the graded sensation across the entire arm under different voltages.

Aside from the parameters of the circuit, the geometry of the electrode making contact with human skin also plays a crucial role in determining the intensity of the haptic perception. Figure 4A shows the minimum capacitance required to elicit a faint haptic perception across various electrode spacings and breakthrough voltage levels. Wider electrode spacings, which usually lead to higher skin impedance, tend to diminish the tactile response. For this reason, the study adopts the smallest possible electrode spacing to maximize stimulation effectiveness. Moreover, stimulation sensitivity varies with the age of the users. Thus, the combined effects of age and electrode position on sensation were examined. By adjusting the breakdown threshold voltage of the GDT, this study evaluated the capacitance *C*_2_ required to achieve a comfortable sensation among different age groups, as shown in Fig. 4 (B and C). From the results, it can be seen that the younger group has a more pronounced haptic perception to the electrotactile property of the GDT, and they can achieve an appropriate level with less energy release, due to the increased skin impedance with age (*57*).

Last, the influence of electrode position was also studied. Figure 4D presents a sensitivity map of all stimulation sites on the participant’s arm. In this study, 22 electrode points were strategically placed across the entire arm, as shown in fig. S8. By controlling the breakdown voltage of the GDT and adjusting the value of storage capacitors, the tactile sensation of different electrode position in the same arm was investigated, where a different feeling was sensed in different positions under the same releasing energy. In detail, the threshold values for all 22 points were acquired from each volunteer and used to generate distribution maps for different sensation levels and volunteers via natural interpolation methods. A notable increase in sensitivity along the outer side of the arm can be observed, which reveals that stimulation intensity often amplifies when electrodes are positioned near blood vessels. It should be noted that at the breakdown voltage threshold of 90 V, even with an increased capacitance to microfarads, participants were still unable to perceive painful stimuli, rendering this figure unsuitable. Furthermore, individual variations in electrical stimulation responses were notable, as depicted in the sensory mapping chart of another participant shown in fig. S9.

### Applications of the SPETH glove: Prosthesis sensation, gesture recognition, and therapy

**Fig. 5.**
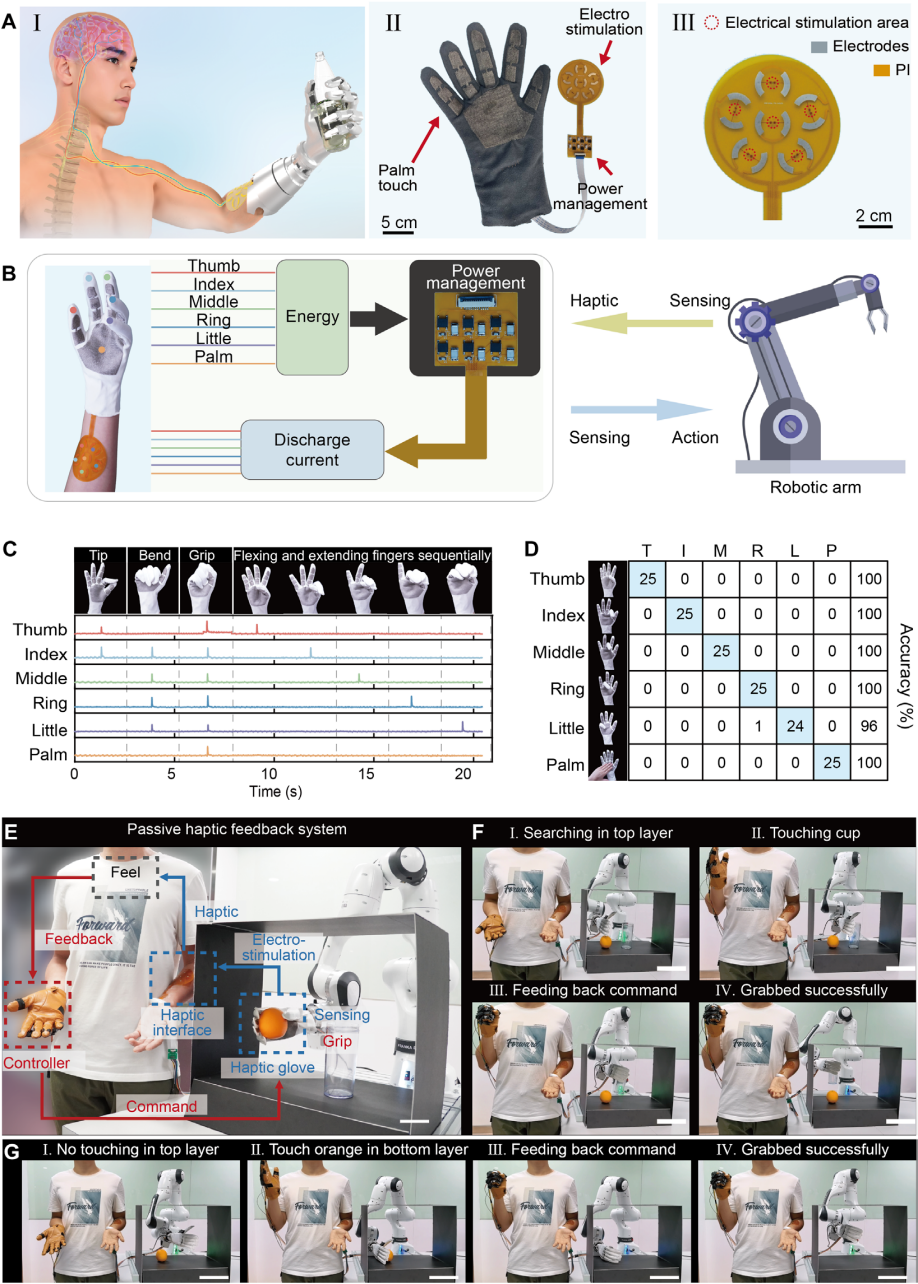
Application of SPETH glove in phantom limb sensation and the passive haptic feedback system. (**A**) [(A), I] The illustration of the haptic glove applied in limb sensation, where the contact between the robotic hand and the target can electrically stimulate tactile feed back to the person’s arm. The photograph of [(A), II] the haptic glove and [(A), III] electrodes. (**B**) The haptic glove consisted of a triboelectric glove, a power management unit, and electrodes. (**C**) Display of the signals generated in different finger gestures. (**D**) Confusion matrix of recognizing different finger gestures. (**E**) The overall illustration of the SPETH glove–based passive haptic feedback system, where the haptic glove worn on the robotic hand can provide a sense of touch to the person’s arm, allowing human control of the robotic hand through this sensory feedback. (**F** and **G**) The demonstration of passive haptic feedback system application in a black box, where the cup and orange with different heights were sensed and grabbed successfully.

This technology enables haptic gloves used in limb sensory feedback, where contact between a prosthetic hand and an intended target generates electrotactile haptic feedback to the user’s arm (Fig. 5A-I). Figure 5A-II shows the photo of the SPETH glove, which is crafted through an embroidery technique on fabric to create TENG electrodes, later manually fashioned into a glove and connected with a haptic module. Figure 5A-III shows an enlarged view of the haptic patch that is based on a flexible printed circuit board; more information on processing can be found in fig. S10. In the limb sensory feedback demonstration, limited by the output of the TENG, we developed six TENG sets for the fingers and the palm, which capture and store energy from interactions with objects (Fig. 5B). The discharge current triggered by the GDT then delivers targeted stimuli, enabling precise sensory perception and feedback. To demonstrate the generated current more intuitively, we have connected light-emitting diodes (LEDs) with electrode positions. When the glove is given mechanical input or actively touches other objects, the LEDs light up (movie S2). In addition, we tested different electrotactile signal patterns during various finger movements, encompassing operations like pinch with the thumb and index finger and the sequential opening and closing of four fingers, illustrated by pulse current diagrams in Fig. 5C. The results demonstrate the glove’s capability to provide tactile sensing and haptic feedback at the same time. A confusion matrix depicting stimulation across different hand regions is presented in Fig. 5D, where three participants discriminated between stimulated glove locations successfully, showcasing precise spatial distinction. The discharge current induced during this process can be used in electrostimulation treatments, explored through electromyography and sensation studies, as shown in fig. S11. Our design also has potential applications in VR and AR. Specifically, in movie S3, we demonstrate the use of our SPETH gloves for grasping virtual objects, highlighting their potential in immersive environments.

The SPETH glove also shows the potential application of a self-powered passive tactile feedback glove in prosthetics. As shown in Fig. 5E, the glove is worn on a robotic hand, with its electrodes positioned on the participant’s right arm to deliver touch feedback. Detecting the arm’s stimulation signals allows the wearer to initiate grasping commands through the left-hand control glove, enabling the robotic arm to autonomously grasp objects. This demonstration underscores how the glove enhances sensory perception for amputees or individuals experiencing tactile degradation. Figure 5 (F and G) portrays the applications of the passive tactile feedback system in a black box setting. Initially, as depicted in Fig. 5 (F to I), the robotic arm scans for elevated items within the box; touching a high cup (Fig. 5F-II) triggers electric stimuli on the participant’s arm, informing them of the object’s contact. The participant then raises his hand to signal object perception (Fig. 5F-III) and issues the grasp command (Fig. 5F-IV), completing the perceptual-feedback cycle. In Fig. 5G, the robotic arm scans the upper layer, finding no objects (Fig. 5G-I), before probing the lower layer, where the participant encounters an orange, prompting arm stimulation and signaling object perception (Fig. 5G-II), culminating in the command to grasp the object (Fig. 5G-III), which is then grasped successfully (Fig. 5G-IV), thus concluding the perception-feedback sequence. This process is demonstrated in movie S4.

## DISCUSSION

In conclusion, this work successfully developed a SPETH glove that can serve as a wearable haptic device based on the triboelectric effect and GDT breakdown to deliver precise electrical stimulation for self-powered haptic feedback. This glove operates with remarkable efficiency, using mere microjoules of energy to induce notable haptic perception. The glove can harness microjoules of energy to produce notable tactile sensations. Representing a substantial leap forward in wearable haptic technology, the SPETH glove seamlessly combines self-sustainability, portability, and cost-effectiveness. Its ability to provide customizable tactile sensations opens up a broad spectrum of potential applications, from enhancing AR and VR experiences to transforming rehabilitation therapies and human-robot interfaces. Our findings underscore the transformative potential of self-powered tactile interfaces and pave the way for a more immersive and engaging future in interactive technologies.

## MATERIALS AND METHODS

### Fabrication of the electrotactile haptic glove

Haptic gloves are constructed from commercially available woven fabric, incorporating conductive pads made of embroidered conductive silver fiber and using FEP as the dielectric layer. The FEP film, with a thickness of 25 μm, is sourced from a single-sided adhesive tape, which can be directly affixed to the fabric electrode. The architecture of the textile-based TENG unit is shown in Fig. 1C-I. The embroidery is performed with Tajima Sai automatic embroidery, and the detailed process is depicted in fig. S1. The final assembly of the glove involves hand sewing.

### Fabrication of the hydrogel-based electrostimulation patch

First, the polyacrylamide (PAAm) ionic conductive hydrogel was fabricated based on previous research (*16*). Initially, a solution of 8 M LiCl, 2 M acrylamide (AAm), and PAAm (with a PAAm:AAm weight ratio of 0.142) was dissolved in deionized water at 60°C for 3 hours. Then, the cross-linker *N*,*N*′-methylenebisacrylamide was added at a concentration of 0.6 wt % of AAm, along with the photoinitiator Irgacure 1173 at a concentration of 1.6 wt % of AAm. The mixture was stirred overnight. Afterward, the gel was poured into a mold and exposed to ultraviolet light for 30 min to ensure complete cross-linking of the hydrogel. Last, the resulting hydrogel, with a thickness of 2 mm, was meticulously attached to the electrode for electrostimulation purposes.

### Robot control

The robotic system includes a force-sensitive Franka Research 3 arm (FRANKA EMIKA) with a five-finger robotic hand RH56BFX-2R (HINYEUNG LIMITED). Initially, the robotic arm scans the contents of a black box to make first contact with the object and stops. At this point, our SPETH glove generates triboelectricity to direct an electric discharge current that stimulates the participant’s arm. If the participant feels such sensation, he could raise his hand to indicate that he has received stimulation and then make a fist to command the robotic hand to grasp the item and retrieve it.

### Characterization and measurements

The electrical performance of TENG and discharge current in (Fig. 2, E and G to I) were measured by Keysight DSOX2014A. The data in Fig. 2 (C and D) were measured by Keithley 6514. The signals generated by different finger gestures (Fig. 5C) were detected by a multimeter system (PowerLab 16/35, AD Instruments). The statistical results of the user study data in Fig. 3 and Fig. 4 were plotted using Python, and Keithley 6514 was also used for auxiliary measurement during the process. The data plots were processed by Origin Lab 2019. Threshold current heatmaps were processed by customized programs in Python. All optical photos were taken by a digital camera (Sony A6400).

### User study

In the threshold study, experiments were conducted with 15 male and 15 female participants to assess various capacitance levels and GDT discharge thresholds at the center of their arms. For the study targeting comfort thresholds across different age groups, three males and three females from each age bracket (20 to 30, 30 to 35, and more than 35) were recruited. To explore tactile perception along the entire arm, 22 specific locations were identified and marked to map graded sensations comprehensively (see fig. S6).

Throughout the experiment, participants were instructed to thoroughly cleanse their arms and ensure relaxation before proceeding with the testing. The discharge voltage and capacitor settings of the haptic system were adjusted until volunteers experienced a defined sensation, after which the intensity was gradually increased. Participants were intentionally kept unaware of the electrical parameters and asked to describe the intensity of their sensations subjectively using a predetermined five-level sensory scale—from “no feeling,” indicating no sensation, to “weak,” representing a very faint stimulus, “appropriate,” which conveys a clearly noticeable sensation, “slightly uncomfortable,” indicating mild discomfort that is still bearable, and “painful,” reflecting overwhelming discomfort that is intolerable. Testing of the device and data collection were performed with the informed consent of all volunteers. All human experiments were performed in accordance with protocols approved by the Human Subjects Ethics Sub-Committee of Research Committee, City University of Hong Kong, Hong Kong, China [Result of Research Ethics Review Application (Human Research)-(HU-STA-00000850)] and conducted in compliance with the guidelines.
