# Supplementary material for: Self-powered electrotactile textile haptic glove for enhanced human-machine interface
Source: Sci Adv. 2025 Mar 21;11(12):eadt0318. doi: 10.1126/sciadv.adt0318 (PMC11927614; doi:10.1126/sciadv.adt0318)
Supplement: Supplementary file 1 — Supplementary Text S1 to S6 Figs. S1 to S11 Table S1 Legends for movies S1 to S4 [file sciadv.adt0318_sm.pdf]

Science Advances
AAAS

Supplementary Materials for

Self-powered electrotactile textile haptic glove for enhanced human-machine interface

Guoqiang Xu *et al.*

Corresponding author: Yunlong Zi, ylzi@hkust-gz.edu.cn; Zijian Zheng, zijian.zheng@polyu.edu.hk; Xinge Yu, xingeyu@cityu.edu.hk

Sci. Adv. **11**, eadt0318 (2025)
DOI: 10.1126/sciadv.adt0318

The PDF file includes:

Figs. S1 to S11
Supplementary Text S1 to S6
Table S1
Legends for movies S1 to S4

Other Supplementary Material for this manuscript includes the following:

Movies S1 to S4

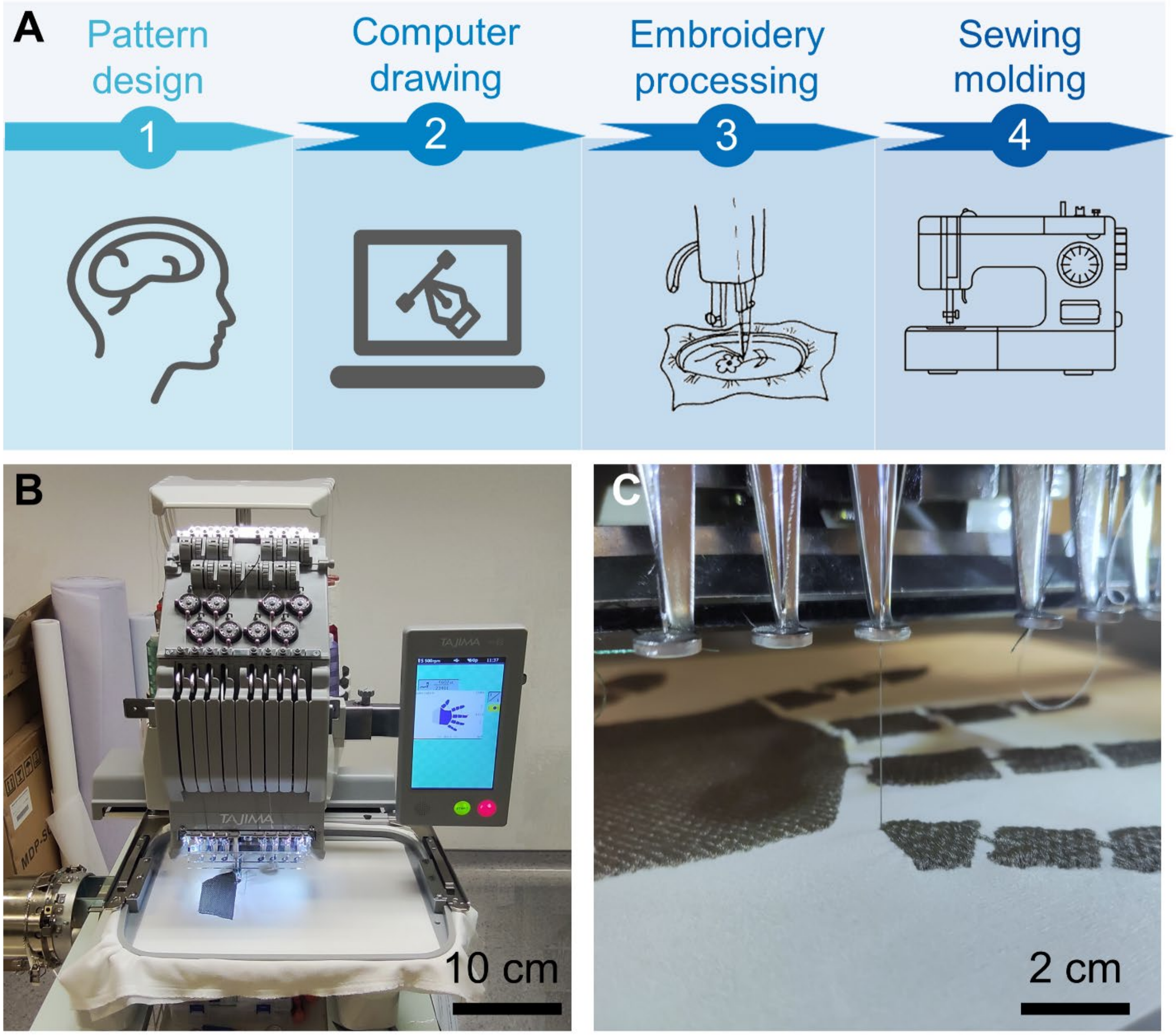

Supplementary Fig. 1 Design and processing of smart wearable gloves. (A) Flowchart of the processing of the smart wearable glove. **(B)** The Tajima Sai automatic embroidery machines were utilized in the creation of specified conductive patterns. **(C)** The enlarge image of the embroidery part.

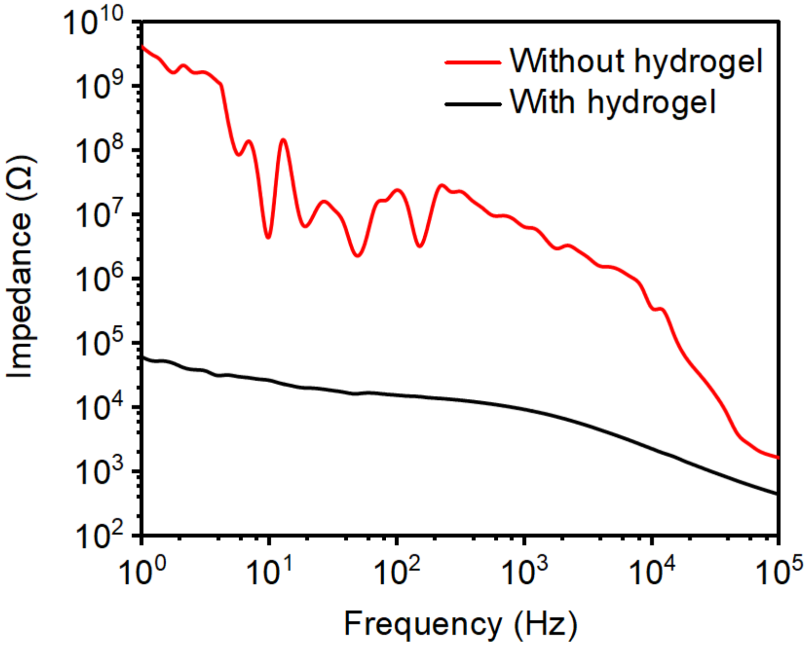

Supplementary Fig. 2 Skin impedance with/without conductive hydrogel.

Text S1: The output performance of TENG unit in SPETH glove

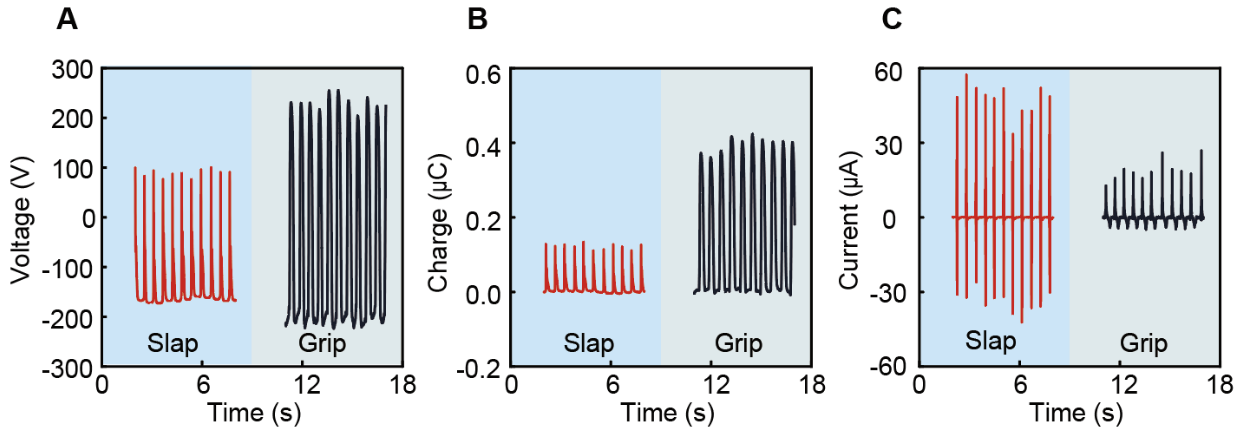

Supplementary Fig. 3 The output performance of TENG unit in SPETH glove. (A) Output voltage of SPETH glove in slap and grip mode; **(B)** Quantity of transferred charge of SPETH glove in slap and grip mode; **(C)** Output current of SPETH glove in slap and grip mode.

Fig. S3 presents the output performance of the TENG unit in SPETH glove. **Fig. S3A**, **Fig. S3B**, and **Fig. S3C** depict the voltage, transferred charge, and current outputs measured during slapping and grasping process with the gloves, respectively. This result indicates that the output during grasping process is higher, with values reaching 250 V, 0.4 μC, and 30 μA. In contrast, the output during slapping process registers approximately 100 V, 0.14 μC, and 60 μA. It is worth noting that the higher current observed during slapping is attributed to the increased speed of this action.

Text S2: Study of the current and transferred charge of SPETH glove with different capacitance and GDT

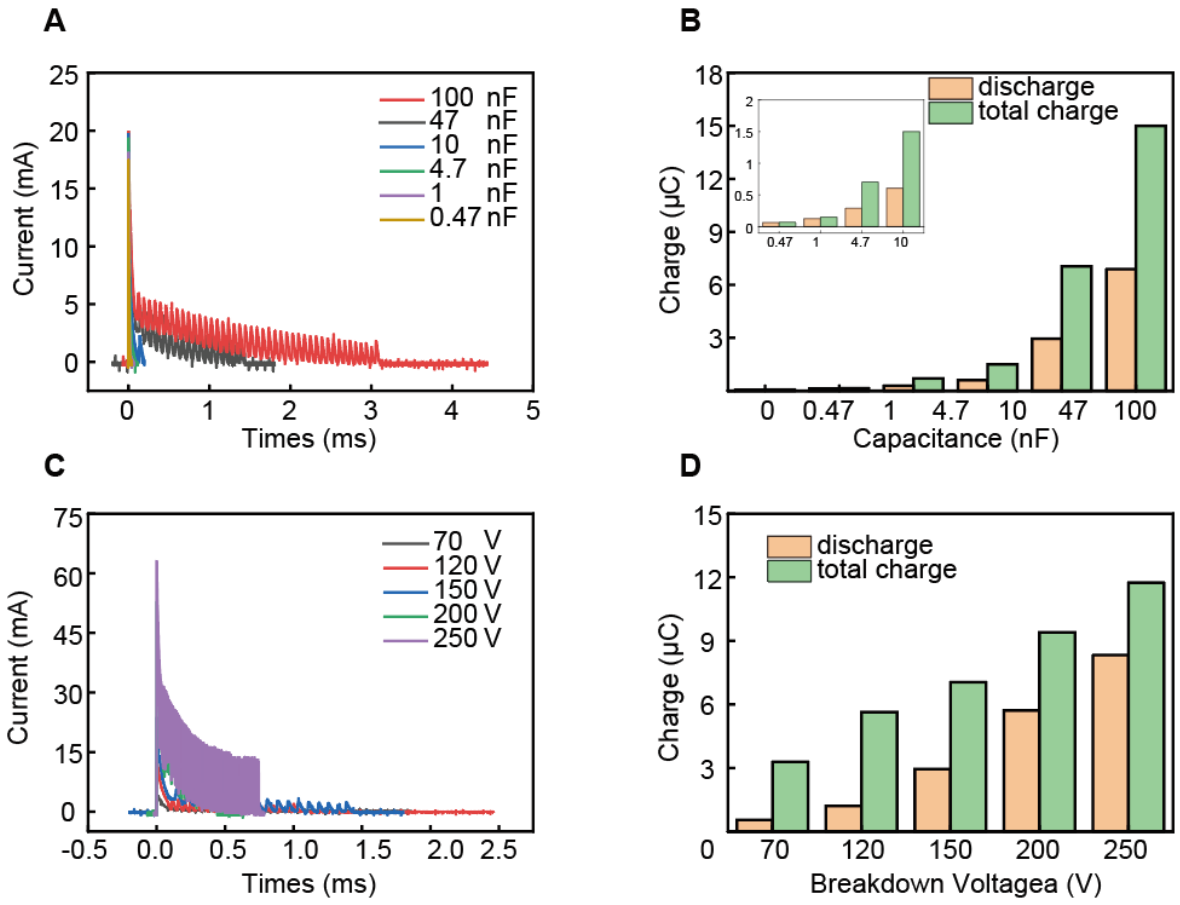

Supplementary Fig. 4 Study of the current and transferred charge of SPETH glove with different capacitance and GDT. (A) Current curves of SPETH glove with a GDT with a breakdown voltage of 150 V under different capacitances; **(B)** Quantity of transferred charge of the SPETH glove with a GDT with a breakdown voltage of 150 V under different capacitances; **(C)** Current curves of SPETH glove with a GDT with a capacitance of 47 nF under different GDT voltage breakdown threshold; **(D)** Quantity of transferred charge of SPETH glove with a GDT with a capacitance of 47 nF under different GDT voltage breakdown threshold.

Fig. S4 presents the testing results of current and transferred charge of SPETH glove with different capacitance and GDT. Specifically, **Fig. S4A** examines the effects of different capacitance values on the pulse current under a fixed breakdown voltage of 150 V, while **Fig. S4B** shows the ratio of the discharge charge through the skin to the total charge stored in the capacitor, indicating that the actual discharge charge through the skin is much less than the total charge stored in the capacitor. Additionally, **Fig. S4C** and **Fig. S4D** respectively display the discharge current curve and the discharge charge amount at a fixed capacitance of 47nF, demonstrating that the percentage of discharge charge significantly increases with the rise in breakdown voltage. However, the breakdown voltages and capacitance values typically used in actual applications are generally small, resulting in a very limited proportion of the charge per discharge.

Text S3: optical image showcase a volunteer's forearm before and after undergoing electrostimulation treatment

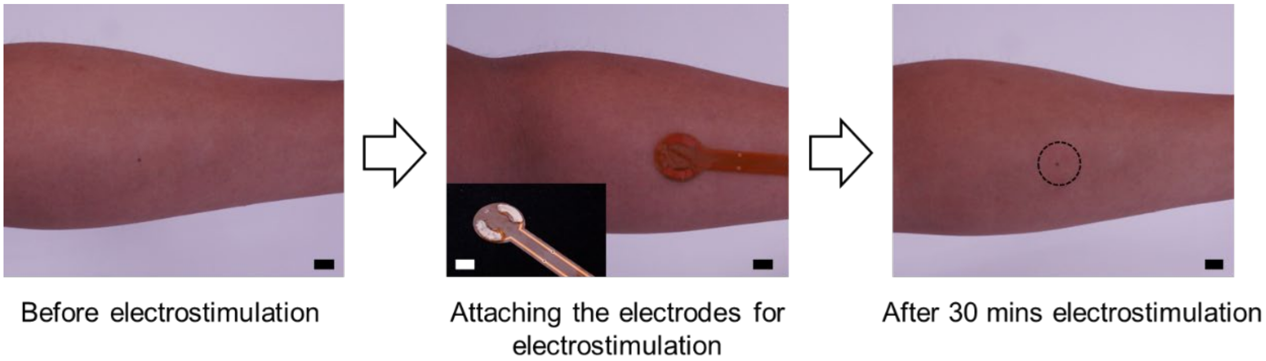

Supplementary Fig. 5 Optical image of the forearm skin changes after electrostimulation. (Scale bar: 1 cm)

Fig. S5 presents the optical image showcase a volunteer's forearm before and after undergoing electrostimulation treatment. The observations indicate an absence of any indications of skin irritation, such as redness or swelling, within the treatment area after 30 minutes of application. Additionally, no residue remained after the device was removed, demonstrating the safety in electrostimulation treatment.

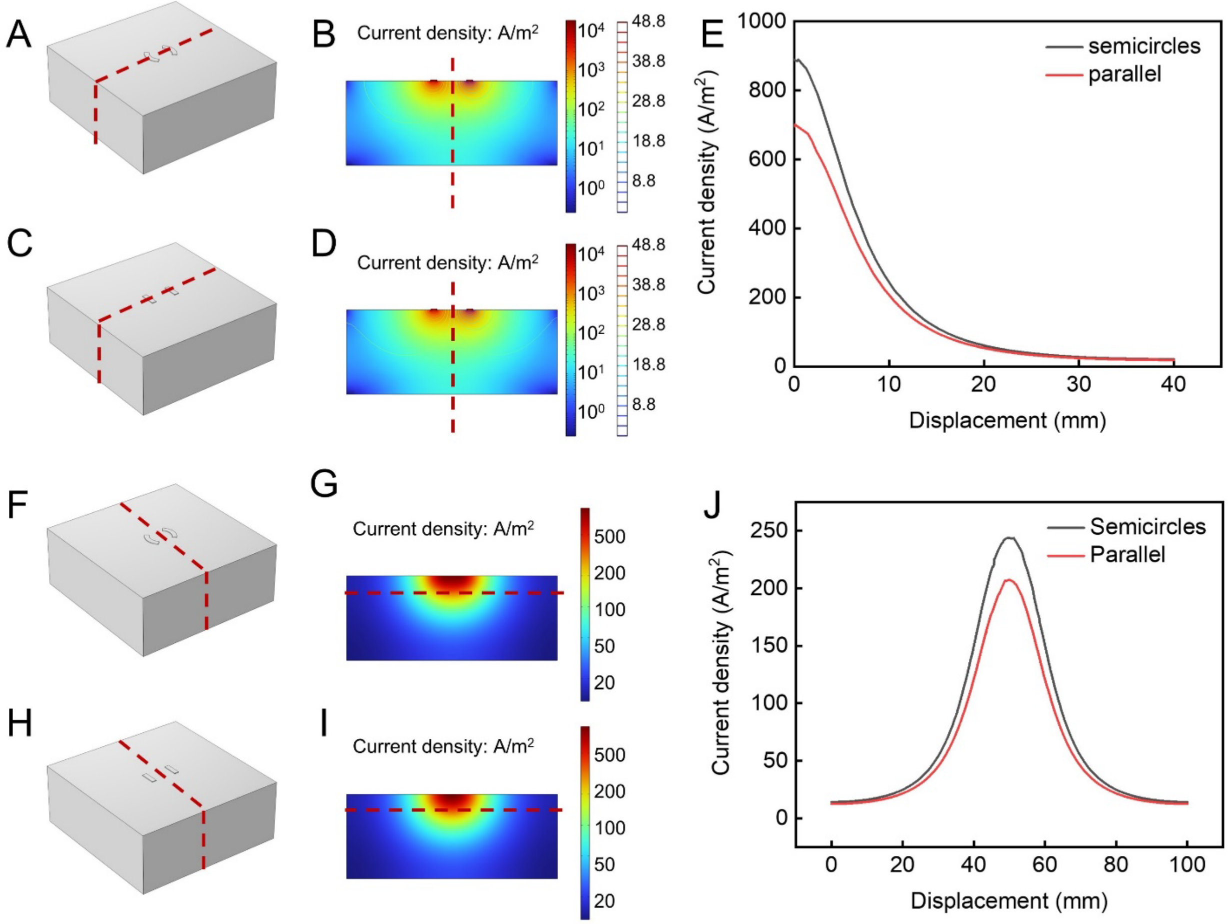

Supplementary Fig. 6 Simulation of Electrode Geometry. (A) 3D model of semicircular electrodes and skin. (B) Current density distribution corresponding to the section marked by the red dashed line in panel A. (C) 3D model of parallel electrodes and skin. (D) Current density distribution corresponding to the section marked by the red dashed line in panel C. (E) Variation of current density with depth, referencing the positions of the dashed lines in panels B and D. (F) 3D model of semicircular electrodes and skin. (G) Current density distribution corresponding to the section marked by the red dashed line in panel F. (H) 3D model of parallel electrodes and skin. (I) Current density distribution corresponding to the section marked by the red dashed line in panel H. (J) Variation of current density with depth, referencing the positions of the dashed lines in panels G and I.

The semicircular shape helps create a deeper and wider current distribution area on the skin, preventing current concentration in a localized area. To illustrate this point, we have included Figure S6, which simulates and compares the differences between semicircular electrodes and parallel electrodes, keeping all other parameters such as electrode thickness and applied voltage consistent. Figures S6A and S6C show 3D models of both electrode types in relation to the skin. We calculated the current distribution along a cross-section, presented in Figures S6B and S6D. Additionally, Figure R2E illustrates the current distribution along the depth at the positions indicated by the red dashed lines in Figures S6B and S6D. The results show that the semicircular electrodes yield higher current values at the same depth, suggesting a more pronounced stimulation effect. Similarly, we provided current distribution data along another cross-section in Figures S6F-I and calculated the current distribution at a depth of 10 mm, displayed in Figure S6J. This analysis demonstrates that the semicircular electrodes generate a larger area of higher current density. Based on these findings, we conclude that the semicircular design is advantageous.

Text S4: Measurement and analysis of the management circuit in a haptic interface

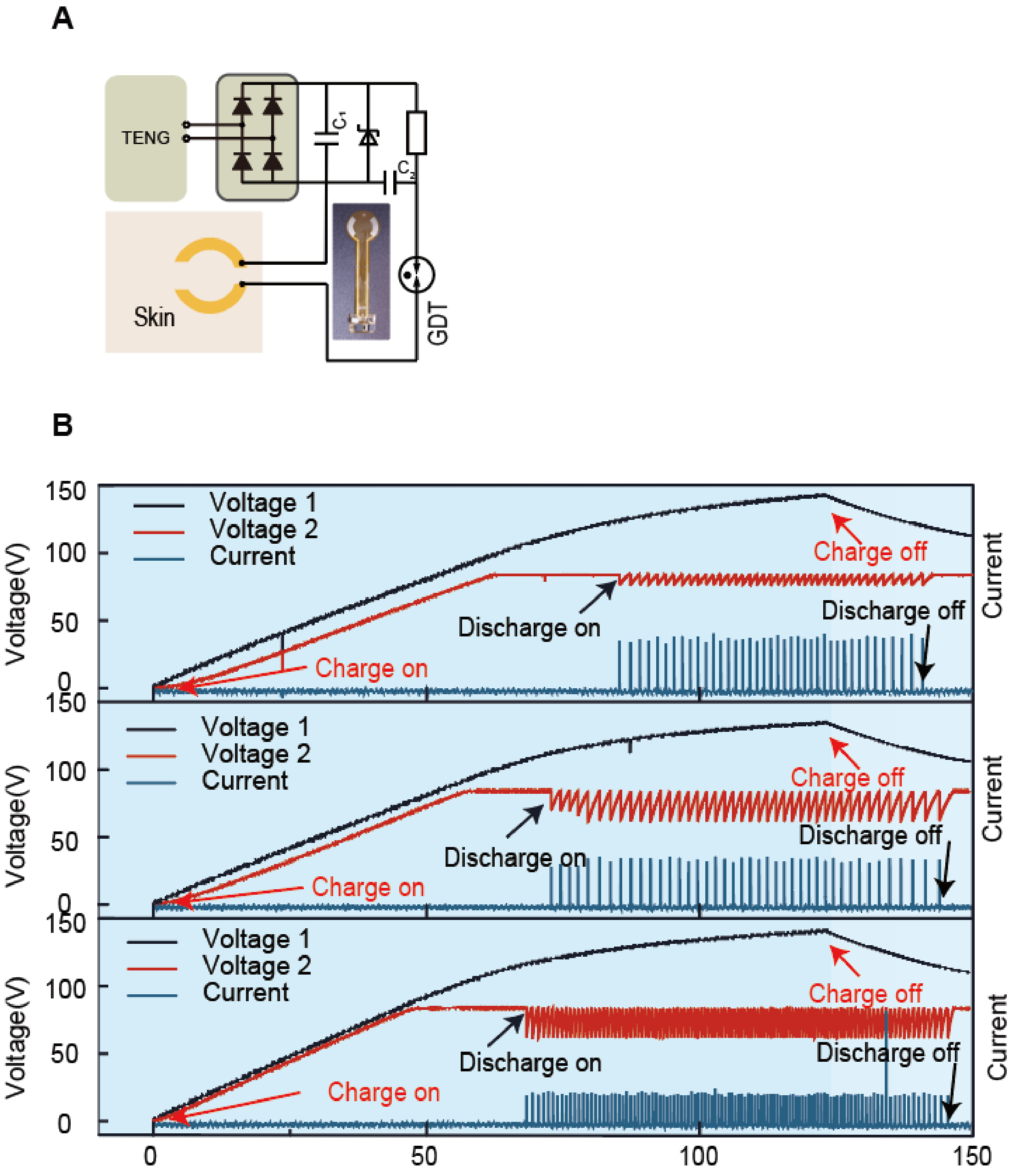

Supplementary Fig. 7 Measurement and analysis of the management circuit in a haptic interface. (A) Schematic diagram of the management circuit. **(B)** Voltage and discharge current curves of SPETH glove with a GDT during discharge in continuous working stages.

Fig. S7 illustrates the study and measurement of the management circuit in a haptic interface. As depicted in **Fig. S7A**, the circuit captures energy generated by the TENG unit, stores it in a storage capacitor C_1, and facilitates a breakdown discharge process in a GDT. This process allows the induced current to be transmitted to the skin, thus eliciting tactile sensations. **Fig. S7B** displays the voltage and discharge current curves of the SPETH glove equipped with a GDT during discharge across continuous working stages, highlighting the impact of varying capacitances of capacitor C_2. Specifically, the larger capacitor, C_1, accumulates energy from the TENG and supplies power to the smaller capacitor, C_2, enabling consistent breakdown.

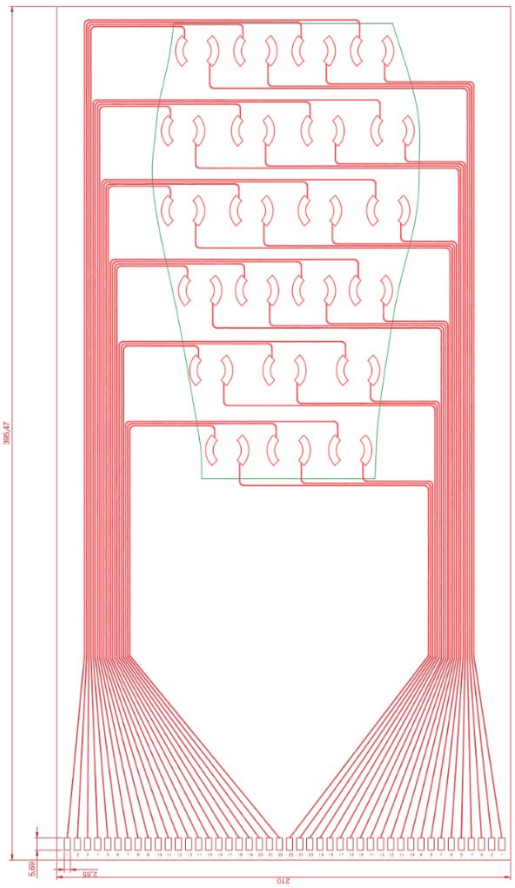

Supplementary Fig. 8 The design of the circuit boards for sensation mapping detection across the entire arm.

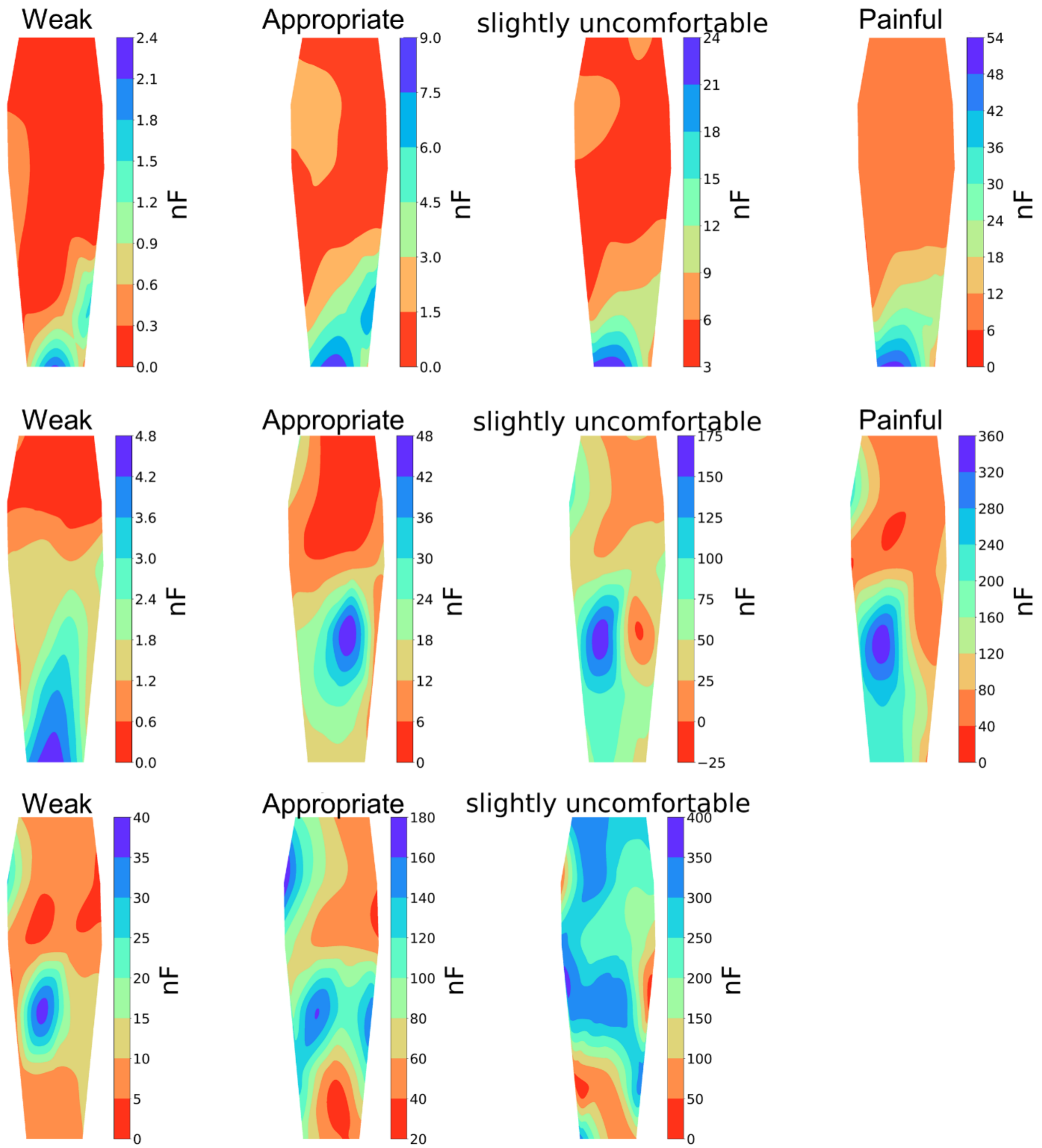

Supplementary Fig. 9 Individual sensation mapping detection results (Volunteer #2)

Text S5: Display images of electrodes, management circuit, and SPETH glove

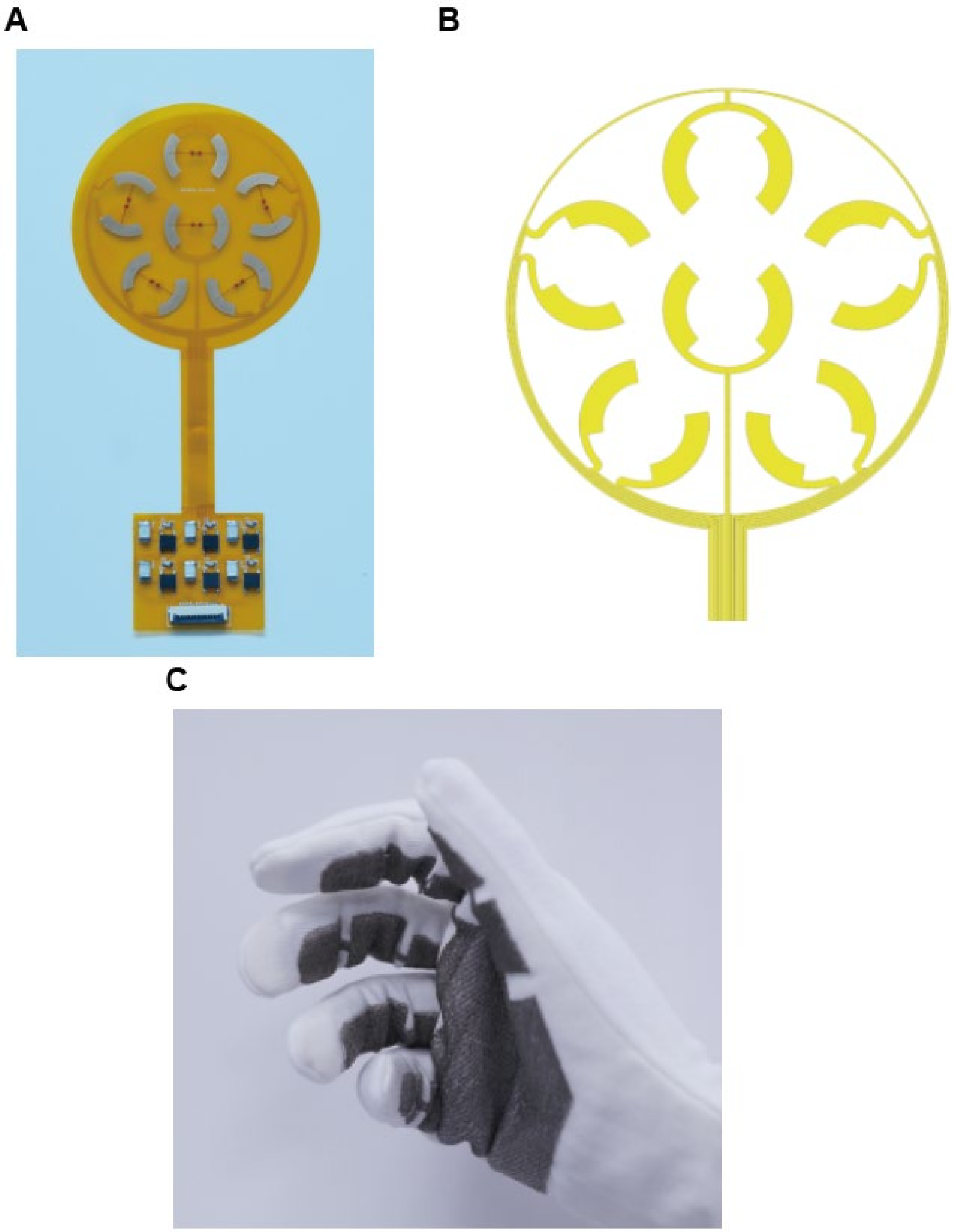

Supplementary Fig. 10 Display images of electrodes, management circuit, and SPETH glove. **(A)** The flexible printed circuit board (FPCB) for passive haptic feedback system; **(B)** Design of electrostimulation electrode; **(C)** Optical image SPETH glove. (Scale bar: 1 cm)

Fig. S10 provides a comprehensive overview of the components of the SPETH glove. **Fig. S10A** presents an optical image of our specially designed FPCB, which includes management circuit with GDT and skin electrode. **Fig. S10B** illustrates the details of electrodes, emphasizing their specific

arrangement. Additionally, **Fig. S10C** displays an optical image of the textile TENG unit of SPETH glove, expertly constructed using commercially available woven fabric. The glove features conductive pads made from embroidered conductive silver fiber and employs FEP as the dielectric layer. Notably, the glove is exceptionally lightweight and exhibits a soft texture, as clearly displayed in **Fig. S10C**.

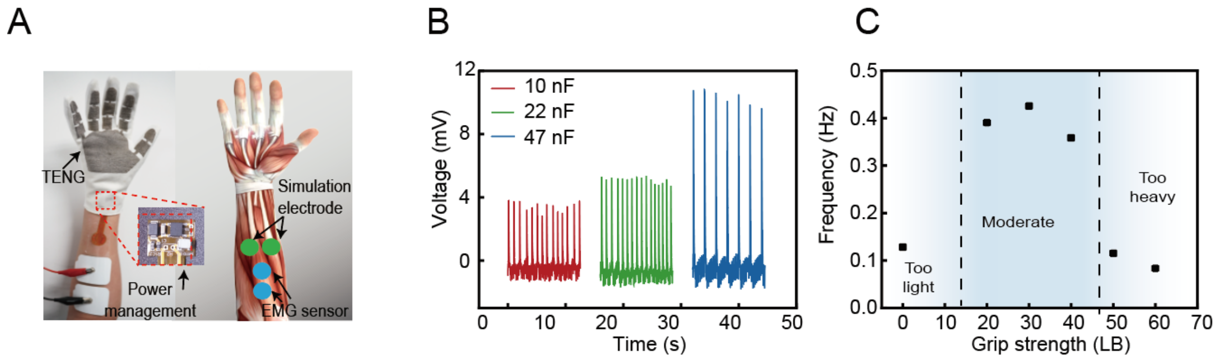

Supplementary Fig. 11 Application of SPETH glove in electrostimulation treatment. (A) The demonstration of haptic glove applied in electrostimulation treatment; **(B)** Electromyography (EMG) curves of the arm during electrical stimulation with the SPETH glove at various capacitances; **(C)** Electrical stimulation discharge frequency of SPETH glove under different grip forces (grip frequency: 1Hz).

Fig. S11 highlights the practical application of the SPETH glove in electrostimulation treatment. **Fig. S11A** depicts a demonstration of the electrostimulation site alongside an electromyography (EMG) sensing demonstration to evaluate the resultant electrical stimulation. The different EMG signal was measured with different capacitance (**Fig. S11B**). Moreover, **Fig. S11C** illustrates how the discharge frequency of electrical stimulation in the SPETH glove varies under different grip strengths at a frequency of 1 Hz, thus showcasing the utility and applicability range of electrical stimulation therapy.

Text S6: comparison of different switch for TENG discharge.

Table S1. Summarizes the advantages and disadvantages of various switch types used for electrical stimulation control of TENG.

Switch Type	Principle	Advantages	Disadvantages
Gas Discharge Tube (GDT)	Gas discharge	✧ Low energy requirements<br>✧ Operates at low voltages<br>✧ Generates pulsed currents<br>✧ Simplified design integration	
Electromagnetic Relay	Gas discharge	✧ Capable of producing strong pulses<br>✧ Operates at low voltages	✧ High energy input requirement<br>✧ Difficulties in additional circuitry to control switch
MOSFET	Field-Effect	✧ Low voltage operation<br>✧ Easy to drive	✧ Reduced effectiveness of stimulation<br>✧ Difficulties in additional circuitry to control switch
MEMS-based Electrostatic Switch	Gas discharge	✧ Low energy requirements<br>✧ Operates at low voltages<br>✧ Generates pulsed currents<br>✧ Flexible structures	✧ Challenges in precise gap fabrication<br>✧ Operational complexity

While several types of switches could be employed—such as electromagnetic relays, MOSFETs, and MEMS-based electrostatic switches—our choice of gas discharge tube (GDT) is informed by several critical factors:

1. **Ease of Drive:** GDT can operate effectively at low energy conditions typical of TENG output, requiring lower currents compared to electromagnetic relays which typically demand higher energy input for activation.
2. **Voltage Requirements:** Unlike other switch types that necessitate higher activation voltages, GDT can achieve breakdown and generate pulsed currents at around tens of volts, making them highly suitable for the textile-based TENG applications we explore.
3. **Pulse Generation:** GDT inherently generate a pulsed current upon breakdown, crucial for creating the distinct stimulation effects needed in our application, whereas other switches such as MOSFETs may not provide as strong of a stimulus due to continuous discharge behavior.
4. **Simplicity of Design:** GDT does not require complex additional circuitry for operation; their activation occurs at a predetermined voltage threshold, simplifying integration into our design.

In contrast, electromagnetic relays, despite their capacity to generate strong pulse currents, are impractical for TENGs due to their high energy requirements, along with the necessity for additional circuitry to control switch operation. Similarly, MOSFETs, while easier to drive, can adversely affect stimulation effectiveness due to their discharge characteristics, requiring supplementary circuitry for control. Lastly, MEMS switches, while promising, face challenges related to precise gap fabrication and operational complexity, as evident from Paschen's law,[4] demanding micro-level control over electrode spacing to achieve breakdown within the desired voltage range, compounded by static attraction effects. In conclusion, we believe that the gas discharge tube is the most viable option for our design due to its ability to operate under the specific

constraints of TENGs effectively, thus providing an optimal solution for our research objectives.

Movie S1.
Fabrication process of SPETH glove

Movie S2.
Illuminating the LED by SPETH glove

Movie S3.
Application of SPETH glove in VR

Movie S4.
SPETH glove-based passive haptic feedback system
